# MiR-146a Contributes to Thromboinflammation and Recurrence in Young Patients with Acute Myocardial Infarction

**DOI:** 10.3390/jpm12071185

**Published:** 2022-07-20

**Authors:** Ascensión M. de los Reyes-García, José Miguel Rivera-Caravaca, Laura Zapata-Martínez, Sonia Águila, Andrea Véliz-Martínez, Nuria García-Barberá, Pablo Gil-Perez, Pedro J. Guijarro-Carrillo, Esteban Orenes-Piñero, Cecilia López-García, María L. Lozano, Francisco Marín, Constantino Martínez, Rocío González-Conejero

**Affiliations:** 1 Servicio de Hematología y Oncología Médica, Hospital General Universitario Morales Meseguer, Centro Regional de Hemodonación, Instituto Murciano de Investigación Biosanitaria (IMIB-Arrixaca), 30003 Murcia, Spain; sregapa@gmail.com (A.M.d.l.R.-G.); laurazap97@gmail.com (L.Z.-M.); sonia.aguila@um.es (S.Á.); nurgarbar@gmail.com (N.G.-B.); pedrojesus.guijarro@gmail.com (P.J.G.-C.); 2 Servicio de Cardiología, Hospital Clínico Universitario Virgen de la Arrixaca, Universidad de Murcia, IMIB-Arrixaca, CIBERCV, 30003 Murcia, Spain; jmrivera429@gmail.com (J.M.R.-C.); andreaveliz19@hotmail.com (A.V.-M.); pablogilperez1@gmail.com (P.G.-P.); cecilopezgarcia23@gmail.com (C.L.-G.); fcomarino@hotmail.com (F.M.); 3 Department of Biochemistry and Molecular Biology, University of Murcia, IMIB-Arrixaca, CIBERCV, 30003 Murcia, Spain; eorenes@um.es; 4 Servicio de Hematología y Oncología Médica, Hospital General Universitario Morales Meseguer, Centro Regional de Hemodonación, Universidad de Murcia, 30003 Murcia, Spain; mllozano@um.es

**Keywords:** miR-146a, acute coronary syndrome, rs2431697, recurrence

## Abstract

Studies on older patients have established notable conceptual changes in the etiopathogenesis of acute coronary syndrome (ACS), but little is known about this disease in young patients (<45 years). Of special interest is thromboinflammation, key at onset, evolution and therapy of cardiovascular pathology. Therefore, we explored whether ACS at an early age is a thromboinflammatory disease by analyzing NETs and rs2431697 of miR-146a (a miRNA considered as a brake of TLR/NF-kB pathway), elements previously related to higher rates of recurrence in atrial fibrillation and sepsis. We included 359 ACS patients (<45 years) and classified them for specific analysis into G1 (collected during the hospitalization of the first event), G2 and G3 (retrospectively collected from patients with or without ACS recurrence, respectively). cfDNA and citH3–DNA were quantified, and rs2431697 was genotyped. Analysis in the overall cohort showed a moderate but significant correlation between cfDNA and citH3–DNA and Killip–Kimball score. In addition, patients with citH3–DNA > Q4 more frequently had a history of previous stroke (6.1% vs. 1.6%). In turn, rs2431697 did not confer increased risk for the onset of ACS, but T carriers had significantly higher levels of NET markers. By groups, we found that cfDNA levels were similarly higher in all patients, but citH3–DNA was especially higher in G1, suggesting that in plasma, this marker may be attenuated over time. Finally, patients from G2 with the worst markers (cfDNA and citH3–DNA > Q2 and T allele) had a two-fold increased risk of a new ischemic event at 2-year follow-up. In conclusion, our data confirm that ACS is younger onset with thromboinflammatory disease. In addition, these data consolidate rs2431697 as a silent proinflammatory factor predisposing to NETosis, and to a higher rate of adverse events in different cardiovascular diseases.

## 1. Introduction

Although modifications of lifestyle and improvements in diagnosis and therapeutics have decreased the incidence of acute coronary syndrome (ACS), in younger patients who present with acute myocardial infarction, this trend is not so clear [1]. The younger population with ACS is a subgroup in which, rather than atherosclerosis, coronary artery embolism, thrombosis anomalies and vessel inflammation, spasm or plaque erosion may be the main trigger of disease [2,3]. Such distinctive features may lead to different presentation, prognosis, progression and treatments [4].

The etiopathogenesis of ACS has undergone notable conceptual changes in recent decades derived from series of older patients. Thus, thrombi heterogeneity may determine the development, the responses to treatments and the outcome [5]. In addition, the concept of plaque rupture has also been conflated with that of plaque erosion, though they are actually two clinically and histologically different processes [3]. Finally, inflammation has emerged as a new element in the onset, in the evolution and as a therapeutic target in cardiovascular pathology [6]. In this new scenario, it is well known that inflammation links both innate immunity and coagulation, in a process known as immunothrombosis [7]. Dysregulation of this process leads into a higher risk of thrombotic complications (thromboinflammation) that take place in sterile inflammatory diseases, such as cardiovascular and autoimmune diseases [8]. Mechanistically, after interacting with platelets, neutrophils migrate to the incipient thrombus and contribute to its propagation through the formation of neutrophil extracellular traps (NETs) that contain a series of molecules (histones, DNA, enzymes) that exert cytotoxic, proinflammatory and prothrombotic effects [9]. All these morphological and phenotypic elements add complexity to cardiovascular diseases to such an extent that the unique natural history model of atherosclerosis is becoming obsolete [10].

Recently, our group has described the participation of an additional element, a microRNA (miRNA), in this system. We reported that miR-146a, which inhibits the TLR/NF-kB pathway and whose expression is regulated by the presence of rs2431697 (among others), has a prognostic role for adverse cardiovascular events in atrial fibrillation [11]. In vitro activation of rs2431697 TT neutrophils also enhances NET release more profusely than C carriers do, revealing a role of miR-146a in NET generation. Moreover, NET markers, together with rs2431697, provided new prognostic information of adverse events in both patients with atrial fibrillation [12] and patients with community-acquired pneumonia [13].

Therefore, it is relevant to analyze ACS in younger patients taking into account these new elements. In the present work, we explored whether ACS at an early age might be a thromboinflammatory disease. Thus, we evaluated the associations between NET markers and the clinical outcomes of ACS in young adults (<45 years), and the relationship between these markers and miR-146a rs2431697 genotype. Finally, we analyzed the prognostic value of these markers in the recurrence of cardiovascular events beyond the onset of the diagnostic event.

## 2. Patients and Methods

### 2.1. Study Population and Patients’ Characteristics

From January 2015 until March 2020, patients with a history of ACS who were under 45 years of age were enrolled. All included patients met the criteria of having suffered a primary ACS event before 45 years of age. In patients with a secondary (or recurrent) ACS event, the inclusion criterion was that the first ACS event had occurred before the age of 45 years. The presence of vascular congenital malformation or traumatic ACS was not found in any of the patients who suffered a secondary ACS event. To reflect “real-life” clinical practice, no other specific exclusion criteria were established.

In order to perform specific analysis, patients were further divided into three different groups. Patients from group 1 (G1) suffered an ACS event when below 45 years of age. Their samples were collected during hospitalization due to the ACS. Patients from group 2 (G2) suffered a first ACS event before 45 years of age and had recurrent ACS thereafter. Samples from G2 were collected within 90 days of the recurrent ACS episode. Finally, patients from group 3 (G3) were stable patients that had an ACS event before 45 years of age and no recurrence. These patients were contacted by telephone and attended a scheduled appointment to draw a blood sample within 90 days of the ACS episode. Additionally, 300 healthy donors were selected as the control group, matched in age and sex with patients.

Written informed consent from all patients and donors was collected, and the study was approved by the Ethics Committee of Hospital General Universitario Morales Meseguer and Hospital Clínico Universitario Virgen de la Arrixaca (Murcia, Spain).

### 2.2. Genomic DNA Extraction

Peripheral blood samples were collected in EDTA tubes. Within 24 h after collection, blood samples were centrifuged at 2500× *g* for 15 min at room temperature (RT). After centrifugation, the buffy coat was isolated for DNA extraction using the QIAcube device with the QIAamp DNA Blood Mini Kit (Qiagen, Madrid, Spain) following the manufacturer instructions. Quantification of total DNA (ng/µL) and quality testing (A260/A280 and A260/A230 ratios) for each sample were performed using a NanoDrop ND-2000 spectrophotometer (Thermo Fisher Scientific, Madrid, Spain). DNA samples were stored at −20 °C until their analysis.

### 2.3. Cell Free DNA Quantification

Cell free double stranded DNA (cfDNA) was quantified in plasma of patients and healthy controls by using a fluorescent nucleic acid stain, Sytox Green reagent (ThermoFisher Scientific, Madrid, Spain). In a 96-well dark plate, the plasma was diluted 1:3 in Tris buffered saline and mixed with an equal volume of 2 µM Sytox Green. The mix was incubated for 15 min in the dark at RT, and then the fluorescence was analyzed at 488 nm for excitation and 528 nm for emission in a plate reader (Biotek Synergy Ht, Izasa, Barcelona, Spain). After the subtraction of autofluorescence, the cfDNA concentration of the sample was calculated by extrapolation of a standard line of known concentrations of DNA from salmon sperm (Sigma-Aldrich, Madrid, Spain).

### 2.4. CitH3-DNA Complexes Quantification

Citrullinated histone H3 and DNA complexes (citH3–DNA) were measured using a sandwich ELISA technique as previously described by Lefrançais et al. [14]. Rabbit anti-citH3 (citrulline R2 + R8 + R17, ab-5103, Abcam, Madrid, Spain) was used as a capture antibody overnight. Then, the wells were washed and blocked, and citrated plasma samples or PBS were added and incubated for 2 h at RT. Next, wells were washed again and incubated with peroxide-conjugated anti-DNA antibody (Cell Death Detection ELISA Kit, Roche Applied Science, Indianapolis, IN, USA) for 2 h at RT. Finally, the optical density (OD) was measured at 405 nm two hours after the addition of the peroxidase substrate, ABTS (ThermoFisher Scientific, Madrid, Spain).

### 2.5. miR-146a Genotyping

miR-146a SNP rs2431697 was genotyped using a commercial Taqman probe (Thermo Fisher Scientific, Madrid, Spain). The reaction was optimized to a final volume of 5 µL, using 0.2 µL of probe (C_26633319_10), 2.5 µL of Premix ExTaq 2x buffer (Thermo Fisher Scientific, Madrid, Spain) and 0.3µL of DNase-free water. This water was also used as a negative control replacing DNA. The assay was carried out in a LightCycler 480 (Roche Farma, Madrid, Spain).

### 2.6. Follow-Up and Endpoints

After collection, all patients were followed-up for up to 2 years according to the standard of care at each routine visit to the outpatient clinic. During this period, all adverse events were recorded. For the present study, the primary endpoints were all-cause mortality, ischemic events (including ST-elevation myocardial infarction (STEMI), non-ST-elevation myocardial infarction (NSTEMI) and stent thrombosis), cardiovascular hospitalization and any cardiovascular outcome. The investigators identified, confirmed and recorded all clinical outcomes.

### 2.7. Statistical Analysis

Continuous variables are expressed as mean ± standard deviation (SD) or median and interquartile range (IQR) as appropriate, whilst categorical variables are expressed as absolute frequencies and percentages. The Pearson Chi-squared test was used to compare proportions, and differences between continuous and categorical variables were assessed using the Mann–Whitney U test or the Student *t*-test, as appropriate.

Correlations were assessed by using the Spearman’s Rho or the Pearson’s correlation coefficient, as appropriate.

Logistic regression models were used to determine the associations between rs2431697 genotype, NETosis markers and the risk of recurrent ischemic events. Results are reported as odds ratios (ORs) with 95% confidence intervals (CIs).

A *p*-value < 0.05 was accepted as statistically significant. Statistical analyses were performed using SPSS version 25.0 (SPSS, Inc., Chicago, IL, USA) for Windows.

## 3. Results

An overall cohort of 359 ACS patients (316 males, 88%) with a median age of 44 (IQR 40–47) years old was enrolled. Of these, 13 (3.6%) had unstable angina, 212 (59.1%) presented STEMI and 134 (37.3%) had NSTEMI. The main characteristics of these patients are shown in Table 1. There were differences in risk factors between healthy controls and ACS patients, but there were no differences in sex and age. The clinical characteristics of patients in each group are presented in Table 2. As shown, we found no relevant significant differences either in demographic data or in rs2431697 frequencies between groups (Table 2).

### 3.1. NETosis Markers in ACS Patients and Healthy Donors

**Figure 1:**
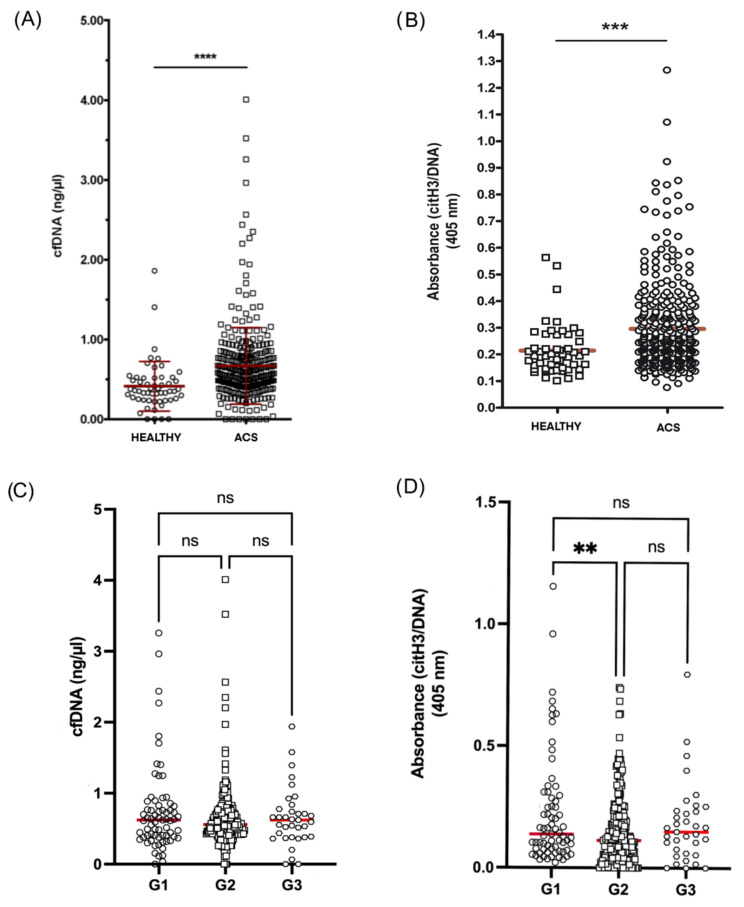
Levels of NETosis markers in ACS young patients. (**A**) cfDNA levels in healthy donors (n = 55) vs. ACS patients (n = 342) measured by Sytox Green fluorescence. (**B**) citH3–DNA complexes in healthy individuals (n = 51) vs. ACS patients (n = 343) measured by ELISA and expressed as relative absorbance at 405 nm. (**C**) cfDNA levels by group of ACS patients (G1 n = 69; G2 n = 238; G3 n = 35). (**D**) citH3–DNA complexes expressed as relative absorbance at 405 nm in the different groups of ACS patients (G1 n = 69; G2 n = 239; G3 n = 35). ns: not significant; ** *p* < 0.01; *** *p* < 0.001; **** *p* < 0.0001.

CfDNA and citH3–DNA complexes in plasma were strongly increased in ACS patients compared to healthy controls (0.668 vs. 0.414 ng/µL (n = 342/n = 55) and 0.296 vs. 0.215 O.D. (n = 343/n = 51), respectively; *p* < 0.0001 for both markers) (Figure 1A,B). We found a significant correlation between cfDNA and citH3–DNA levels (r = 0.389; *p* < 0.0001) (Supplementary Figure S1).

Limiting the analysis to only ACS patients, we found no differences in the cfDNA levels between patient groups (Figure 1C). However, citH3–DNA complexes were significantly higher in group G1 compared to G2 (0.227 vs. 0.148 ng/µL (n = 69/n = 239), *p* < 0.01) (Figure 1D).

### 3.2. Relationship between rs2431697 and ACS

Distribution of rs2431697 genotypes was not statistically different between all ACS and controls (Table 1). That distribution was in agreement with Hardy–Weinberg equilibrium in both groups (ACS: *p* = 0.695; controls: *p* = 0.424). Analysis of these frequencies in different subgroups of ACS patients discarded any influence of the genotype in the ACS onset (Table 2).

### 3.3. rs2431697 and NETosis Markers in ACS Patients

**Figure 2:**
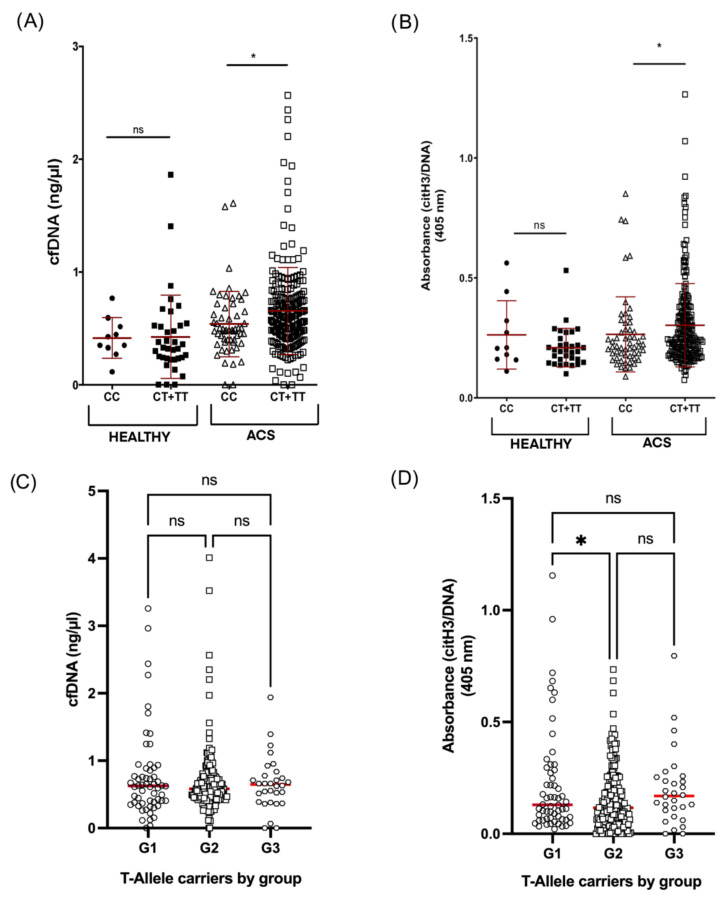
Levels of NETosis markers in young ACS patients by genotype. (**A**,**B**) cfDNA levels and citH3–DNA complexes levels in healthy donors or ACS patients considering the dominant model of rs2431697 genotype, CC (n = 56) vs. CT and TT (n = 231). (**C**,**D**) cfDNA levels and citH3–DNA complexes levels in those patients carrying the T-allele of rs2431697 in G1 (n = 58), G2 (n = 196) and G3 (n = 30). ns: not significant; * *p* < 0.05.

We explored in all ACS patients associations between genetic and allelic frequencies with cfDNA and citH3–DNA complexes (Supplementary Figure S2A,B, respectively) and found no significant differences between genotypes. However, significant differences were found for the dominant model, since levels in T allele carriers (n = 231) were higher than in C homozygous patients (n = 231) (0.654 vs. 0.540 ng/µL; *p* = 0.015 and 0.304 vs. 0.265 O.D.; *p* = 0.041, respectively). No significant association was observed between the rs2431697 genotype and any of the two markers in healthy controls (Figure 2A,B).

When analyzing patients by groups, again we found significant differences in citH3–DNA complexes levels between group G1 and G2 (0.224 vs. 0.154 O.D. (n = 58/n = 196), *p* < 0.05), but none in the cfDNA levels or between other groups (Figure 2C,D).

### 3.4. Associations between NETosis Markers and the Outcome of ACS

In the overall cohort of ACS patients, we found positive and moderate correlations between cfDNA and citH3–DNA levels and the Killip–Kimball score (Pearson’s r = 0.201, *p* < 0.001 and r = 0.173, *p* = 0.002, respectively). This score allows establishing a prognosis of the evolution and the probabilities of death in the first 30 days after the infarction. Supporting the association between NETs markers and clinical status, we also found that patients with levels of citH3–DNA complexes above Q4 (OD > 0.219) had higher prevalence of stroke history vs. the rest of the patients (5/82 (6.1%) vs. 4/250 (1.6%), *p* = 0.026) (Supplementary Figure S3).

### 3.5. Predictive Ability of NETosis Markers Combined with rs2431697 for Cardiovascular Outcomes

Finally, we analyzed if the T allele of the rs2431697 genotype, in addition to NET markers, identified patients with a higher risk for recurrent ischemic events. For this aim, patients were followed up for two years. In this analysis, we considered patients at higher risk to be those who were carriers of the T allele of rs2431697 and had the combination of NET markers levels above the median (cfDNA > 0.573 ng/µL and citH3–DNA OD > 0.116, respectively).

Thus, the risk of new ischemic events was not significantly increased in patients fulfilling these criteria from the overall cohort (OR: 1.23; 95% CI: 0.72–2.12, *p* = 0.452) (Table 3). However, patients from G2 (i.e., those who already had a recurrent ACS) presented a higher risk of recurrent ischemic events for the combination of markers considered (OR: 2.09; 95% CI: 1.10–3.97, *p* = 0.024) (Table 3).

## 4. Discussion

Since Mangold et al. described that the load of NETs in culprit coronary thrombi is significantly related to the size of the infarcted area and to ST-segment resolution [15], many other groups have shown that the components of NETs have a dual utility in cardiovascular diseases, as potential therapeutic targets and as biomarkers that might define the biology of the cardiovascular event [6,16]. As far as we know, there are no data about the relationship between circulating NETs-related components and the clinical outcomes in young ACS. We here investigated the thromboinflammatory background in young patients by evaluating plasma cfDNA and citH3–DNA complexes and rs2431697 genotype in relation to the onset and recurrence of cardiovascular events.

### 4.1. NETs Markers in Plasma from Young ACS

Our data showed that cfDNA and citH3–DNA complexes in plasma were significantly increased in ACS patients when compared to healthy controls. These data strongly suggest that NETosis is significantly increased in young ACS subjects in comparison with matched controls, although we were not able to determine if NETs were the cause or consequence of the events, since all the samples were quantified post-ACS. The utility of NET markers in cardiovascular disease is controversial, since several factors may influence the interpretation of their presence: the compartment where they are quantified, their specificity as NET markers and the time elapsed since the event. Regarding the locations where they are measured, there seems to be a consensus that all NET markers are elevated at the site of the lesion, whereas contradictions are found in peripheral locations. Thus, Stakos et al. compared intracoronary samples from normal and pathological angiographies and found elevated NET markers only in the latter [17]. It has been described that NETosis markers were at higher levels in coronary circulation than in plasma in STEMI patients [18,19]. The specificity of measured NETosis markers (cfDNA, MPO, citrullinated histone H3, MPO-DNA or citH3–DNA complexes) might add a level of confusion. The most specific MPO-DNA or citH3–DNA might lose usefulness away from the injured zone [18]. Thus, it has been suggested that these markers within different locations would indicate differences in degrees of inflammation between the thrombus and the periphery [20]. Our results suggested that both cfDNA and citH3–DNA complexes could work as indicators of NETosis in plasma in younger ACS. Thus, we speculate that ACS before the age of 45 years could be the consequence of a premature inflammatory background that predisposes to NETosis. In older patients, aging and inherent polypharmacy could be contributing to the lack of clarity in the usefulness of plasma NET markers.

### 4.2. Validity of NET Levels beyond the Acute Event

Regarding the stability of cfDNA and citH3–DNA levels over time from the first ACS event, our data agree with those reported in series of older patients. Indeed, it has been reported that cfDNA, although non-specific for NETosis, behaves as a stable marker [21], and our data from young ACS showed that cfDNA levels were similar in the different ACS groups. By contrast, our data suggest that citH3–DNA, even remaining higher than in healthy subjects, could decrease over time in young ACS, since they were significantly higher in G1 in comparison to the rest of the patients. Similar results were reported for the TATICS-TIMI18 trial, since MPO levels associated with a higher risk of recurrence of ischemic event at 30 days, but this association was lower at 6 months [22].

### 4.3. Association between ACS, NETosis and rs2431697 Genotype

Next, we investigated whether the miR-146a rs2431697 genotype is related to ACS in young patients. Although Wang et al. reported that rs2431697 genotype is a risk factor for coronary artery disease in Chinese patients [23], our data showed that this SNP is not related to the onset of ACS in our patients. Differences in genetic background together with demographical and acquired risk factors might account for this discrepancy. Interestingly, ACS patients that had the T allele had significantly higher cfDNA and citH3–DNA levels than CC patients. Therefore, we here demonstrated for the first time that rs2431697 is related to NETosis in plasma in ACS young patients. We already described this association in the worse evolution of patients with atrial fibrillation [12] and in cardiovascular complications of patients with community-acquired pneumonia with sepsis [13]. Consequently, we propose that rs2431697 may be a genetic thromboinflammatory marker of cardiovascular complications of any inflammatory disease. However, this genetic marker would need to be “primed”, so that the sum of inflammatory stimuli (e.g., classical cardiovascular risk factors, overrepresented in patients vs. controls) will provoke the dysregulation that would reveal the functional effect of this SNP. In this sense, our data showed that rs2431697 had no effect on plasma NET markers in healthy controls. Moreover, we previously described that monocytes from healthy volunteers carrying TT genotype transcribed more *IL6* mRNA than CC after LPS activation [11]. Supporting this, the hypothesis that miRNA exerts a functional effect in a pathological context has been previously postulated [24].

### 4.4. Clinical Outcomes, NET Markers and rs2431697 Genotype

Finally, we further evaluated relationships between thromboinflammatory markers and clinical outcomes. Our data showed a moderate but significant association between higher NET plasma markers and severity, since both cfDNA and citH3–DNA levels were significantly associated with the Killip–Kimball score corresponding to the index ACS. Similarly, higher cfDNA in plasma has been significantly associated with shortened long-term survival after STEMI [20,21], but citH3–DNA was not [20]. In addition, it has been reported that high cfDNA in plasma correlates with more specific parameters, such as peak TnT or infarct size, a few days after percutaneous coronary intervention [15,25] but not later [20]. In this sense, in young ACS patients, we found the most significant associations between NET plasma markers and Killip–Kimball score in G1 patients, suggesting that, as described in older patients, the time elapsed from the event to the collection of the sample can be decisive. Interestingly, young patients with the highest levels of the specific NET marker (citH3–DNA > Q4) had suffered a stroke before ACS more frequently than the rest. Thus, we proposed that NET plasma markers might still have prognostic value that is being disturbed by technical, methodological and design difficulties.

When we analyzed the occurrence of new cardiovascular events, our data showed that those patients from G2 with high NET markers in plasma carrying rs2431697-T allele, had a significantly higher risk of recurrent ischemic events. These results suggest a role for this SNP in thromboinflammation in younger ACS patients and are in accordance with similar findings described by our group in atrial fibrillation [11,12] and sepsis [13] patients.

In conclusion, our data suggest that in young patients, as in older patients, thromboinflammation is a pathologic state underlying ACS. On the one hand, NETosis markers in plasma seem to be useful indicators of onset in younger patients. On the other hand, we here described for the first time that rs2431697 could be an additional risk factor conferring a worse prognosis to younger ACS patients. We speculate that rs2431697-T establishes a silent proinflammatory status that upon reaching the appropriate threshold predisposes one to greater NETosis, promoting a higher rate of adverse cardiovascular events. Further studies in larger samples of young ACS patients are required to confirm our results and to translate them to older patients.

## Figures and Tables

**Table 1:** Age, sex, main clinical characteristics and rs2431697 genotype of the ACS cohort vs. healthy donors.

	Healthy Donors (n = 300)	ACS Patients (n = 359)	*p*-Value
Age, mean (range)	42 (22–45)	44 (40–47)	0.152
Male sex, N (%)	251 (83.7)	316 (88.0)	0.115
STEMI, N (%)	-	212 (59.1)	-
NSTEACS, N (%)	-	147 (40.9)	
NSTEMI, N (%)	-	134 (37.3)	-
Unstable angina, N (%)	-	13 (3.6)	-
Obesity, N (%)	25 (8.3)	124 (34.5)	<0.0001
Hypertension, N (%)	26 (8.7)	218 (60.7)	<0.0001
Diabetes, N (%)	6 (2.0)	74 (20.6)	<0.0001
Dyslipidemia, N (%)	61 (20.3)	288 (80.2)	<0.0001
Current smoking, N (%)	41 (13.7)	126 (35.1)	<0.0001
rs2431697 genotype			
CC, N (%)	53 (17.7)	60 (18.7)	0.689
CT, N (%)	141 (47.0)	154 (42.9)	0.329
TT, N (%)	71 (23.7)	105 (29.2)	0.106
C-allele, N (%)	194 (64.7)	214 (59.6)	0.183
T-allele, N (%)	212 (70.7)	261 (81.3)	0.563

NSTEACS = non-ST segment elevation acute coronary syndromes; NSTEMI = non-ST segment elevation myocardial infarction; STEMI = ST segment elevation myocardial infarction. Genotype was available in 265 controls and in 321 ACS.

**Table 2:** Age, sex, main clinical characteristics and rs2431697 genotype of the ACS patients by groups.

	G1 (n = 79)	G2 (n = 244)	G3 (n = 36)	*p*-Value
Age, mean ± SD	42 (39–44)	45 (42–49)	41.5 (39–43)	0.305
Male sex, N (%)	63 (79.7)	218 (89.3)	35 (97.2)	0.052
STEMI, N (%)	44 (55.7)	140 (57.5)	28 (77.8)	0.053
NSTEACS, N (%)	35 (44.3)	104 (42.5)	8 (22.2)	
Obesity, N (%)	21 (26.6)	79 (32.4)	13 (36.1)	0.603
Hypertension, N (%)	21 (26.6)	64 (26.2)	6 (16.7)	0.412
Diabetes, N (%)	10 (12.6)	33 (13.5)	5 (13.9)	0.922
Dyslipidemia, N (%)	25 (31.6)	82 (33.6)	14 (38.9)	0.814
Current smoking, N (%)	50 (63.3)	177 (72.5)	28 (77.8)	0.316
rs2431697 genotype				
CC, N (%)	12 (15.2)	43 (19.8)	5 (15.6)	0.745
CT, N (%)	34 (43.0)	102 (42.1)	19 (52.8)	0.749
TT, N (%)	26 (32.9)	72 (29.8)	8 (22.2)	0.683
C-allele, N (%)	46 (58.2)	145 (59.9)	24 (66.7)	0.907
T-allele, N (%)	60 (83.3)	174 (80.2)	27 (84.4)	0.642

NSTEACS = non-ST segment elevation acute coronary syndromes; STEMI = ST segment elevation myocardial infarction. Genotype was available in G1 = 72, G2 = 217 and G3 = 32.

**Table 3:** Associations of combination of cfDNA, citH3–DNA complexes and rs2431697 with the risk of recurrent ischemic events.

Patient Group	Criteria	OR; 95% CI *	*p*-Value
Overall cohort	citH3–DNA > Q2 + cfDNA> Q2 + rs2431697 T allele	1.23; 0.72–2.12	0.452
G1	citH3–DNA > Q2 + cfDNA> Q2 + rs2431697 T allele	0.38; 0.04–3.48	0.391
G2	citH3–DNA > Q2 + cfDNA> Q2 + rs2431697 T allele	2.09; 1.10–3.97	0.024
G3	citH3–DNA > Q2 + cfDNA> Q2 + rs2431697 T allele		

* Risk of recurrence at 2 years of follow-up. Median (Q2) citH3–DNA OD = 0.116. Median (Q2) cfDNA = 0.573 ng/μL.

## Data Availability

Not applicable.

## Supplementary Materials

The following supporting information can be downloaded at: https://www.mdpi.com/article/10.3390/jpm12071185/s1, Figure S1: Correlation between citH3-DNA and cfDNA. Levels of citH3-DNA and cfDNA were measured in plasma from all ACS patients and Pearson’s correlation analysis was performed using SPSS software; Figure S2: Levels of NETosis markers in ACS young patients. A) cfDNA levels in healthy donors (n = 55) vs. ACS patients (n = 342) measured by Sytox Green fluorescence. B) citH3-DNA complexes in healthy individuals (n = 51) vs. ACS patients (n = 343) measured by ELISA and expressed as relative absorbance at 405 nm; Figure S3: Percentage of patients with previous stroke in relation with citH3-DNA levels. ACS patients with previous stroke with levels of citH3-DNA below Q4 quartile were compared with those above Q4. * *p* < 0.05.

## References

[1] Gupta A., Wang Y., Spertus J.A., Geda M., Lorenze N., Nkonde-Price C., D’Onofrio G., Lichtman J.H., Krumholz H.M. (2014). Trends in acute myocardial infarction in young patients and differences by sex and race, 2001 to 2010. J. Am. Coll. Cardiol..

[2] Rubin J.B., Borden W.B. (2012). Coronary heart disease in young adults. Curr. Atheroscler. Rep..

[3] Kolte D., Yonetsu T., Ye J.C., Libby P., Fuster V., Jang I.K. (2021). Optical Coherence Tomography of Plaque Erosion: JACC Focus Seminar Part 2/3. J. Am. Coll. Cardiol..

[4] Gulati R., Behfar A., Narula J., Kanwar A., Lerman A., Cooper L., Singh M. (2020). Acute Myocardial Infarction in Young Individuals. Mayo Clin. Proc..

[5] Alkarithi G., Duval C., Shi Y., Macrae F.L., Ariëns R.A.S. (2021). Thrombus Structural Composition in Cardiovascular Disease. Arterioscler. Thromb. Vasc. Biol..

[6] Stark K., Massberg S. (2021). Interplay between inflammation and thrombosis in cardiovascular pathology. Nat. Rev. Cardiol..

[7] Engelmann B., Massberg S. (2013). Thrombosis as an intravascular effector of innate immunity. Nat. Rev. Immunol..

[8] Pircher J., Engelmann B., Massberg S., Schulz C. (2019). Platelet-Neutrophil Crosstalk in Atherothrombosis. Thromb. Haemost..

[9] Fuchs T.A., Brill A., Duerschmied D., Schatzberg D., Monestier M., Myers D.D., Wrobleski S.K., Wakefield T.W., Hartwig J.H., Wagner D.D. (2010). Extracellular DNA traps promote thrombosis. Proc. Natl. Acad. Sci. USA.

[10] Pasterkamp G., den Ruijter H.M., Giannarelli C. (2022). False Utopia of One Unifying Description of the Vulnerable Atherosclerotic Plaque: A Call for Recalibration That Appreciates the Diversity of Mechanisms Leading to Atherosclerotic Disease. Arterioscler. Thromb. Vasc. Biol..

[11] Roldán V., Arroyo A.B., Salloum-Asfar S., Manzano-Fernández S., García-Barberá N., Marín F., Vicente V., González-Conejero R., Martínez C. (2014). Prognostic role of MIR146A polymorphisms for cardiovascular events in atrial fibrillation. Thromb. Haemost..

[12] Arroyo A.B., de los Reyes-García A.M., Rivera-Caravaca J.M., Valledor P., García-Barberá N., Roldán V., Vicente V., Martínez C., González-Conejero R. (2018). MiR-146a Regulates Neutrophil Extracellular Trap Formation That Predicts Adverse Cardiovascular Events in Patients With Atrial Fibrillation. Arterioscler. Thromb. Vasc. Biol..

[13] Arroyo A.B., Fernández-Pérez M.P., del Monte A., Águila S., Méndez R., Hernández-Antolín R., García-Barberá N., de los Reyes-García A.M., González-Jiménez P., Arcas M.I. (2021). miR-146a is a pivotal regulator of neutrophil extracellular trap formation promoting thrombosis. Haematologica.

[14] Lefrançais E., Mallavia B., Zhuo H., Calfee C.S., Looney M.R. (2018). Maladaptive role of neutrophil extracellular traps in pathogen-induced lung injury. JCI Insight.

[15] Mangold A., Alias S., Scherz T., Hofbauer T., Jakowitsch J., Panzenböck A., Simon D., Laimer D., Bangert C., Kammerlander A. (2015). Coronary Neutrophil Extracellular Trap Burden and Deoxyribonuclease Activity in ST-Elevation Acute Coronary Syndrome Are Predictors of ST-Segment Resolution and Infarct SizeNovelty and Significance. Circ. Res..

[16] Döring Y., Libby P., Soehnlein O. (2020). Neutrophil Extracellular Traps Participate in Cardiovascular Diseases: Recent Experimental and Clinical Insights. Circ. Res..

[17] Stakos D.A., Kambas K., Konstantinidis T., Mitroulis I., Apostolidou E., Arelaki S., Tsironidou V., Giatromanolaki A., Skendros P., Konstantinides S. (2015). Expression of functional tissue factor by neutrophil extracellular traps in culprit artery of acute myocardial infarction. Eur. Heart J..

[18] Liu J., Yang D., Wang X., Zhu Z., Wang T., Ma A., Liu P. (2019). Neutrophil extracellular traps and dsDNA predict outcomes among patients with ST-elevation myocardial infarction. Sci. Rep..

[19] Hofbauer T.M., Ondracek A.S., Mangold A., Scherz T., Nechvile J., Seidl V., Brostjan C., Lang I.M. (2020). Neutrophil Extracellular Traps Induce MCP-1 at the Culprit Site in ST-Segment Elevation Myocardial Infarction. Front. Cell Dev. Biol..

[20] Langseth M.S., Helseth R., Ritschel V., Hansen C.H., Andersen G.Ø., Eritsland J., Halvorsen S., Fagerland M.W., Solheim S., Arnesen H. (2020). Double-Stranded DNA and NETs Components in Relation to Clinical Outcome After ST-Elevation Myocardial Infarction. Sci. Rep..

[21] Wang X., Yang D., Liu J., Fan X., Ma A., Liu P. (2018). Prognostic value of culprit artery double-stranded DNA in ST-segment elevated myocardial infarction. Sci. Rep..

[22] Morrow D.A., Sabatine M.S., Brennan M.L., De Lemos J.A., Murphy S.A., Ruff C.T., Rifai N., Cannon C.P., Hazen S.L. (2008). Concurrent evaluation of novel cardiac biomarkers in acute coronary syndrome: Myeloperoxidase and soluble CD40 ligand and the risk of recurrent ischaemic events in TACTICS-TIMI 18. Eur. Heart J..

[23] Wang Y., Wang X., Li Z., Chen L., Zhou L., Li C., Ouyang D.S. (2017). Two Single Nucleotide Polymorphisms (rs2431697 and rs2910164) of miR-146a Are Associated with Risk of Coronary Artery Disease. Int. J. Environ. Res. Public Health.

[24] Mendell J.T., Olson E.N. (2012). MicroRNAs in stress signaling and human disease. Cell.

[25] Helseth R., Solheim S., Arnesen H., Seljeflot I., Opstad T.B. (2016). The Time Course of Markers of Neutrophil Extracellular Traps in Patients Undergoing Revascularisation for Acute Myocardial Infarction or Stable Angina Pectoris. Mediat. Inflamm..

