# Supplementary material for: MiR-146a Contributes to Thromboinflammation and Recurrence in Young Patients with Acute Myocardial Infarction
Source: J Pers Med. 2022 Jul 20;12(7):1185. doi: 10.3390/jpm12071185 (PMC9318357; doi:10.3390/jpm12071185)
Supplement: Supplementary file 1 [file jpm-12-01185-s001.zip › jpm-1767965-supplementary.pdf]

Figure S1.

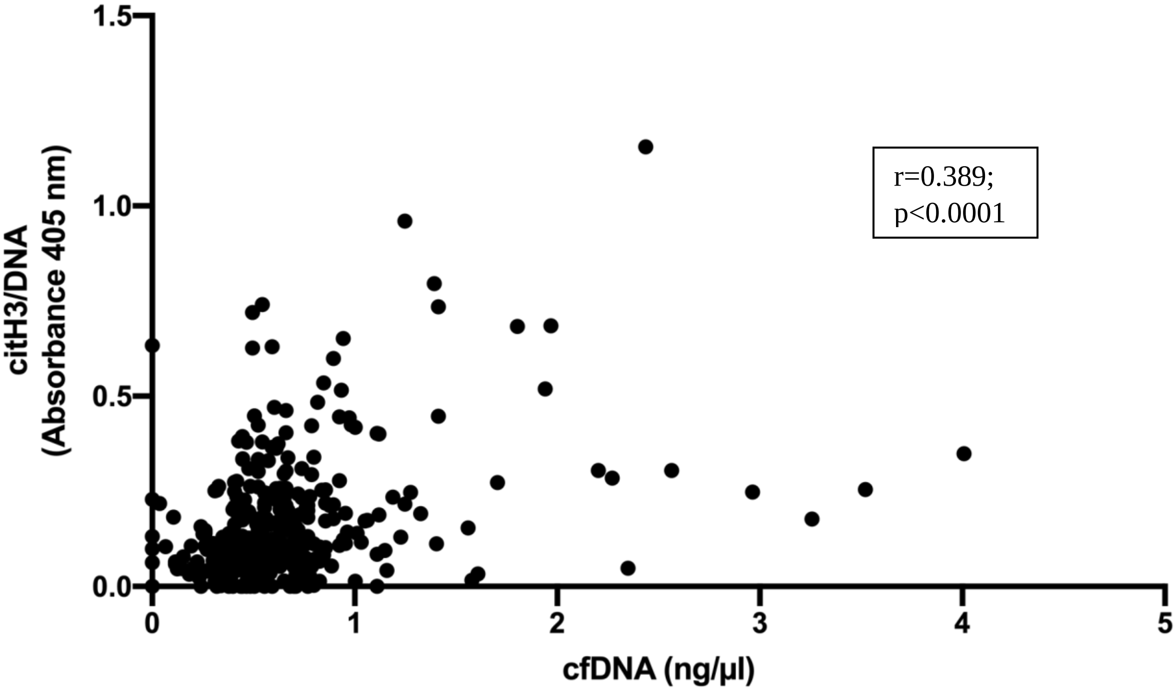

Figure S1. Correlation between citH3-DNA and cfDNA. Levels of citH3-DNA and cfDNA were measured in plasma from all ACS patients and Pearson's correlation analysis was performed using SPSS software.

Figure S2.

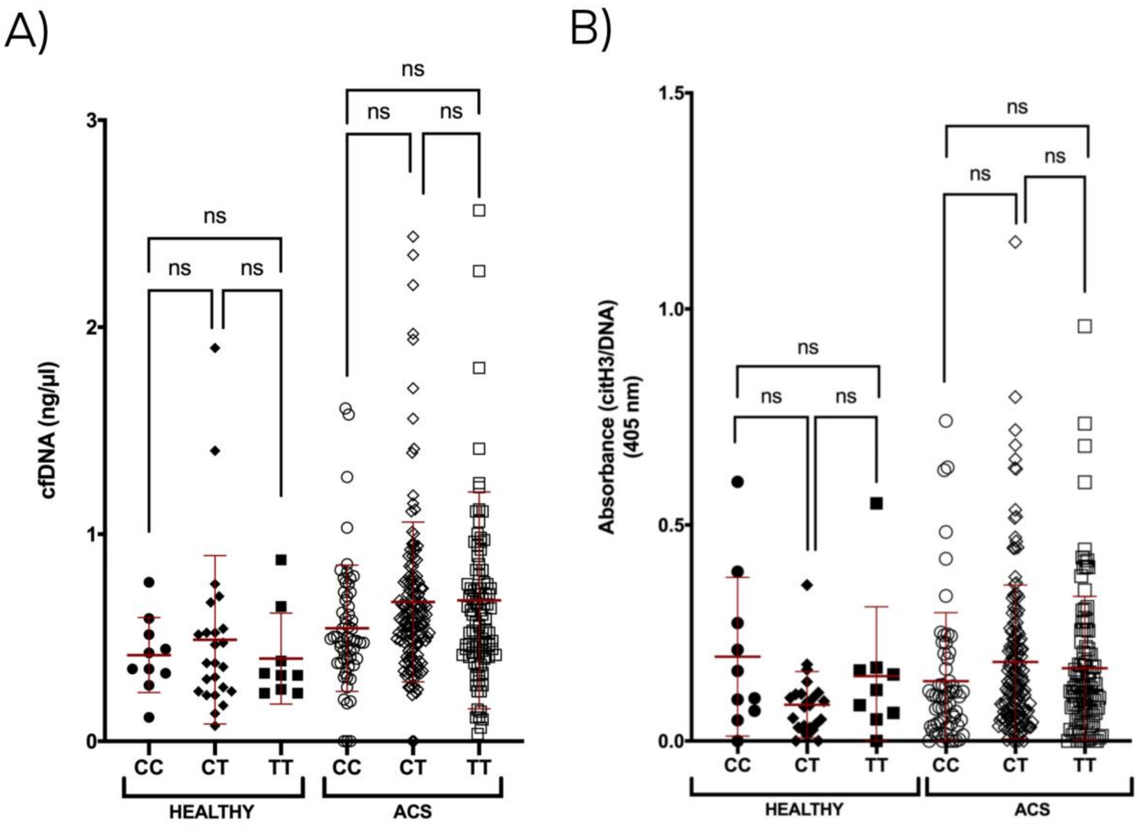

Figure S2. Levels of NETosis markers in ACS young patients. A) cfDNA levels in healthy donors (n=55) vs ACS patients (n=342) measured by Sytox Green fluorescence. B) citH3-DNA complexes in healthy individuals (n=51) vs ACS patients (n=343) measured by ELISA and expressed as relative absorbance at 405 nm.

Figure S3.

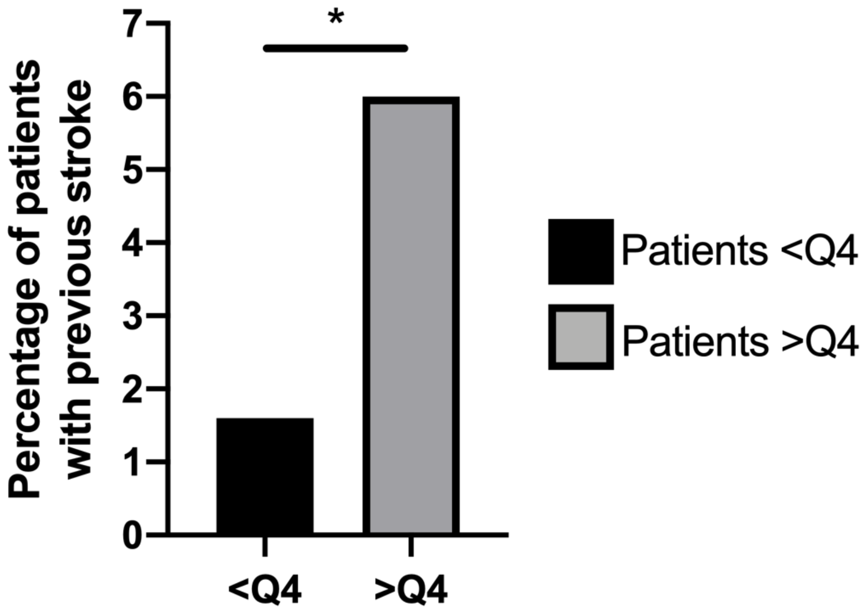

Figure S3. Percentage of patients with previous stroke in relation with citH3-DNA levels. ACS patients with previous stroke with levels of citH3-DNA below Q4 quartile were compared with those above Q4. *p<0.05.
